# Oral dysbiosis induced by *Porphyromonas gingivalis* is strain-dependent in mice

**DOI:** 10.1080/20002297.2020.1832837

**Published:** 2020-10-12

**Authors:** Emile Boyer, Patricia Leroyer, Ludivine Malherbe, Shao Bing Fong, Olivier Loréal, Martine Bonnaure Mallet, Vincent Meuric

**Affiliations:** a INSERM, INRAE, Univ Rennes, CHU Rennes, Institut NUMECAN (Nutrition Metabolisms and Cancer), Rennes, France; b Université de Strasbourg, CNRS, GMGM UMR 7156, Strasbourg, France

**Keywords:** Periodontitis, animal model, mice, *Porphyromonas gingivalis* W83, *P. gingivalis* TDC60, oral microbiota, alveolar bone loss

## Abstract

**Background:** *Porphyromonas gingivalis* strain W83, one of the most widely investigated, is considered virulent in the context of periodontitis. The recently isolated *P. gingivalis* TDC60 has been reported to be highly pathogenic, although it has not yet been investigated in a mouse periodontitis model by oral gavage.

**Aim:** Our aim was to compare the virulence of both strains by evaluating their impact on alveolar bone loss and the composition of oral microbiota.

**Methods:** We inoculated by oral gavage C57BL/6 mice with either one of the two *P. gingivalis* strains and compared to a sham-treated group, without antibiotics pre-treatment. The mandibular alveolar bone of treated mice and controls were assessed, one month after the final inoculation, by microCT measurements. Moreover, at this time, we characterized their oral microbiota by 16S rRNA gene sequencing.

**Results:** While *P. gingivalis* W83 successfully initiated periodontitis, TDC60-treated mice only experienced moderate lesions. Furthermore, only W83-treated mice exhibited a specific distinct microbiota, with significantly lower richness and evenness than other samples, and decreased proportions of taxa usually found in healthy individuals.

**Conclusion:** This association between alveolar bone loss and a major persistent shift of the oral microbiota gives insights into virulence discrepancies among these bacterial strains.

## Introduction

Periodontitis is a chronic inflammatory disease that affects tissues surrounding the teeth. Recent advances in the pathogenesis of periodontitis have suggested that polymicrobial synergy and microbiota dysbiosis together with a dysregulated immune response can induce inflammation-mediated damage in periodontal tissues [1]. *Porphyromonas gingivalis* is still considered as a major driver for the dysbiosis, especially due to its ability to initiate periodontitis in animal models.

Initially applied in a non-human primate model [2], *P. gingivalis* is widely used in murine models of periodontitis induced by oral gavage. Since its first description in 1994, this model allowed to explore the complex interactions between the bacteria and the host immunity [3]. Variations in alveolar bone loss demonstrated the different virulence of several *P. gingivalis* strains in the mouse oral gavage model [4], and one could hypothesize that specific virulent clones of periodontal pathogens may cause more severe periodontitis. The vast majority of animal studies used *P. gingivalis* W50 and W83. These two strains were isolated from clinical samples in the 1950s and have been found to be highly similar in genomic studies [5]. Recently, the *P. gingivalis* TDC60 strain was isolated from a Japanese patient with severe periodontitis in the 2000s [6]. According to the authors, TDC60 displayed a higher pathogenicity than other *P. gingivalis* strains, including W83.

Although TDC60 and W83 both express a majority of the virulence factors specific to *P. gingivalis* (Table 1), they are relatively distant in phylogeny [5]. Moreover, the genome of *P. gingivalis* displays a great variability with functional and metabolic incidence, thus affecting the virulence of the strains [7]. Currently, TDC60 is considered as the most aggressive *P. gingivalis* strain [8]. However, to our knowledge, only the abscess model has been used to assess the pathogenicity of TDC60.

**Table 1.** Virulence factors of *Porphyromonas gingivalis* and their known genetic variation between strains W83 and TDC60. Kgp is a subtype of the proteinase gingipains; FimA is the major protein subunit of the major fimbriae; FimCDE are the accessory proteins of the major fimbriae; Mfa1 is the major protein subunit of the minor fimbriae; RagAB are outer membrane proteins and lipoproteins; the capsule is the capsular polysaccharide antigens (K-antigen).

	*P. gingivalis* W83	*P. gingivalis* TDC60	Functions
Kgp catalytic domain	Type I	Type I	Major proteinase Nutrition Immune evasion
Kgp terminal haemagglutinin	K3	K3	Adhesion to host proteins
FimA	Type IV Poorly fimbriated	Type II	Adhesion to the host cells Bacterial coaggregation
FimCDE	Type I	Type II	Adhesion to the host cells Immune evasion
Mfa1	Disrupted	Type I	Bacterial coaggregation
RagAB	*rag-1*	*rag-4*	Immunogen
Capsule	K1	Unknown	Immune evasion Bacterial coaggregation

Hajishengallis et al. showed that an indigenous bacterial community was required to induce periodontitis, as *P. gingivalis* failed to initiate bone loss in germ-free mice [9]. The authors pinpointed the fact that the disease involved a disruption in the host-microbiota homeostasis caused by *P. gingivalis* inoculation. The discordance in bone loss obtained with different *P. gingivalis* strains could therefore be related to their variable ability to cause such imbalance. However, an extensive characterization of the oral microbiota after inoculation of *P. gingivalis* is lacking, despite the fact that molecular-based approaches to bacterial identification may give an easy access to its taxonomic composition, and could serve as a biomarker of the host homeostasis in the oral cavity.

In this study, our aim was to compare the virulence of W83 and TDC60 *P. gingivalis* strains by evaluating their impact on alveolar bone loss and the composition of the oral microbiota, after chronic inoculation by oral gavage, without antibiotics pre-treatment.

## Methods

### Animals

The study was approved by the ethical committee of Rennes for animal experimentation. All experimented mice were specific pathogen-free male C57BL/6JRj bred and raised in Janvier Labs (Saint-Berthevin, France). The animals were purchased at 6 weeks of age and kept in the animal colony at UMS Biosit in the University of Rennes 1 (Arche), and given free access to tap water and food (Teklad 19% Protein Rodent Diet). The animals were group-housed (three per cage) and maintained under standard conditions of temperature, atmosphere and light. All experimental procedures were performed in agreement with European law and regulations. This study conforms to the ARRIVE guidelines [10].

### Bacteria

*P. gingivalis* strain W83 was directly obtained from the American Type Culture Collection (ATCC) and strain TDC60 was obtained from the Japan Collection of Microorganisms (Riken BioResource Research Center JCM). These strains are maintained at the University of Rennes 1, frozen at – 80°C in cryobeads. For the experiments, the bacteria were transferred and maintained on Columbia 3 agar plates supplemented with 5% (v/v) defibrinated horse blood (bioMérieux, France), 25 mg.L^−1^ of hemin, and 10 mg.L^−1^ of menadione. The cultures were incubated at 37°C in an anaerobic chamber Macs-VA500 (Don Whitley) flooded with 80% N_2_, 10% H_2_ and 10% CO_2_.

### Periodontitis mouse model by oral gavage

Following one-week acclimatization, the experiments started with 7 weeks old animals and were developed according to Baker et al. with slight modifications [3]. Animals were randomly divided into three groups according to their treatment: Control, *P. gingivalis* W83 and *P. gingivalis* TDC60 (*n* = 6 mice/group). Animals were cohoused according to their group to avoid horizontal transmission (3 mice/cage). Prior to each infection, the bacteria were grown in enriched brain-heart infusion (BHI) broth containing, per liter, 37 g of BHI powder (AES Chemunex, France), 5 g of yeast extract (Conda, Dutscher), 25 mg of hemin (Sigma), and 10 mg of menadione (Sigma), placed in an AnaeroPack™ rectangular jar (Mitsubishi Gas Chemical Co. Inc., Tokyo, Japan) with an AnaeroGen™ 3.5 L (Mitsubishi Gas Chemical Co. Inc.) for 24 h at 37°C to reach the mid-exponential phase. The W83- and TDC60-infected groups received 10^9^ CFU of live *P. gingivalis* of the corresponding strain, resuspended in 100 µL of PBS. The infection was made by direct inoculation of the oral cavity, three times a week, during 5 weeks. Controls were sham-infected mice which received the PBS, but no *P. gingivalis*. The oral administrations were made at the same time for all groups.

### Sample collection

Oral swab samples were taken during the third week of the infection phase and stored at – 80°C, before DNA extraction. One month after the final oral gavage, mice were anaesthetised and sacrificed at 16 weeks of age, and mandibles were dissected. One hemi-mandible was quickly frozen in liquid nitrogen and stored at – 80°C, before performing the microbiota analyses. The other hemi-mandible was fixed in 70% ethanol 10% formaldehyde for 24 h at 4°C, and then stored in absolute acetone at 4°C until micro-computed tomography (micro-CT).

### Micro-computed tomography

Micro-CT was performed on the hemi-mandible in the GEROM research unit (Angers, France) with a Skyscan 1272 (Bruker). The samples were placed in microtubes, filled with water to prevent desiccation. The tubes were fixed on brass stubs with plasticine and scanned with the following parameters: 9 µm resolution, X-ray energy of 70 kV and 142 µA for 1.9 s exposure, 0.2° rotation step, 5.000097 μm image pixel size. The data were reconstructed with NRecon (v. 1.7.0.4, Bruker-MicroCT)

### Quantification of alveolar bone loss

The DataViewer software (v. 1.5.6, Bruker-MicroCT) was used to visualise bone and produce the sagittal slices. All images were reoriented such that the cemento-enamel junction (CEJ) and the root apex (RA) appeared [11]. Among the sagittal images, the image which showed the most recession of alveolar bone was selected for measurement. The distance from the alveolar bone crest (ABC) to the CEJ was measured in µm with the CTAn software (v. 1.18.8, Bruker-MicroCT) at two interdental sites: between the first and the second mandibular molars (M1M2) or between the second and the third mandibular molar (M2M3). As described elsewhere, the CEJ-ABC distance was the shortest distance from ABC to the line connecting the adjacent CEJs [11]. Measurements of root lengths from the CEJ to the RA were also taken to assess the percentage of remaining alveolar bone using an equation previously described: Percent remaining bone (%) = ([root length – CEJ-ABC]/root length) × 100 [12].

### Extraction and amplification of bacterial DNA

Total DNA was extracted from frozen oral swabs and hemi-mandibles using the QIAamp DNA Mini Kit (Qiagen, France) according to the manufacturer’s recommendations. The DNA was then kept frozen at – 80°C prior to amplification. DNA from oral swabs was pooled together according to their group, and then amplified to assess the colonization of the administered *P. gingivalis* in the oral cavity by using specific primers for the 16S rRNA gene of the bacterium (forward: 5ʹ-TGG-GTT-TAA-AGG-GTG-CGT-AG-3ʹ; reverse: 5ʹ-CAA-TCG-GAG-TTC-CTC-GTG-AT-3ʹ) [13] with 35 cycles of PCR at an annealing temperature of 57°C. The PCR products were resolved by electrophoresis in 3% agarose gel in Tris-acetate-EDTA buffer. The V3-V4 regions of the 16S rRNA gene were amplified with the primers 338F (5′-ACT-CCT-ACG-GGA-GGC-AGC-AG-3′) and 802R (5′-TAC-NVG-GGT-ATC-TAA-TCC-3′) using 25 amplification cycles with an annealing temperature of 45°C. The PCR products were sequenced with the Illumina MiSeq at the GeT-PlaGe facility (Toulouse, France).

### Oral microbiota analysis

The FASTQ files from the GeT-PlaGe facility are archived at NCBI Sequence Read Archive under the BioProject Accession Number PRJNA667316. The FASTQ files were processed with the QIIME2 software (v. 2018.4, https://qiime2.org/) following a pipeline adapted from the ‘Moving Pictures’ tutorial (https://docs.qiime2.org/2018.4/tutorials/moving-pictures/) [14]. The files were imported as ‘PairedEndFastqManifestPhred33’ format. The pipeline DADA2 was used to control the sequence quality and construct the feature table. The forward and reverse sequences were truncated at 250 bases, with all other parameters set to default. Sequences count per sample ranged between 4,041 and 17,201. Prior to the taxonomic assignation at the genus level, reference reads were extracted from the ‘Ribosomal Database Project’ based on matches to the primers pair (338 F/802 R) and trained as a Naive Bayes classifier (RDP release 11, training set No. 16, http://rdp.cme.msu.edu/misc/rel10info.jsp). The reads that were classified as Archaea or Unassigned were removed from the feature table. The core diversity analysis was performed with a specific sampling depth (4,041 reads). The core diversity included *alpha* (S_obs_, Shannon–Weaver) and *beta* (weighted UniFrac) diversity metrics.

### Statistics

Data were analysed using the R (v. 3.5.0) and RStudio softwares (v. 1.1.383, https://www.rstudio.com/). Non-parametric tests were used and considered significant for *p* < 0.05. The results were expressed as mean ± standard error of the mean. The Kruskal–Wallis with Dunn’s multiple comparisons tests were used to compare means for quantitative data related to weight, alveolar bone parameters, *alpha* diversity indices and taxa relative abundances. The PERMANOVA and ANOSIM tests were performed on *beta* diversity metric with the QIIME2 diversity plugin [15]. A linear discriminant analysis of the taxa relative abundance was computed with the LEfSe algorithm (parameters set to default) in Galaxy (https://huttenhower.sph.harvard.edu/galaxy/). All plots were made with ‘ggplot2ʹ for RStudio [16], except for the LEfSe results and the 3D PCoA plot (EMPeror) [17].

## Results

**Figure 1.**
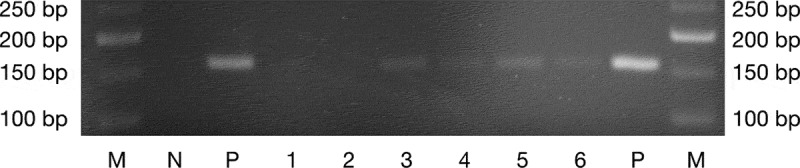
*P. gingivalis* specific 16S rRNA gene amplicons from DNA of oral swabs after electrophoresis on a 3% agarose gel. **1–2**: sham-infected mice (controls); **3–4**: W83-treated mice; **5–6**: TDC60-treated mice; **N**: negative control; **P**: positive control (expected amplicon size: 161 bp). **M**: molecular weight marker (Quick-Load Low Molecular Weight DNA Ladder, NEB®).

The body weight was not significantly different between the three groups (*n* = 6 mice/group) and no adverse event has been observed at any time of the experiments (Table 2). The PCR amplification made on oral swabs taken during the third week of infection confirmed the colonization of the oral cavity of mice by both *P. gingivalis* strains compared to controls (Figure 1).

**Table 2.** Evolution of the animals’ weight during the experiments. Data are presented as mean ± standard error (*n* = 6 mice/group). The *p-*value of the Kruskal–Wallis test is indicated.

	Treatment			
Time of experiment	Controls	TDC60	W83	*p*-value
Baseline (g)	22.17 ± 0.36	23.34 ± 0.47	22.68 ± 0.60	0.29
Sacrifice (g)	26.95 ± 0.62	29.03 ± 0.91	28.23 ± 0.98	0.37

**Figure 2.**
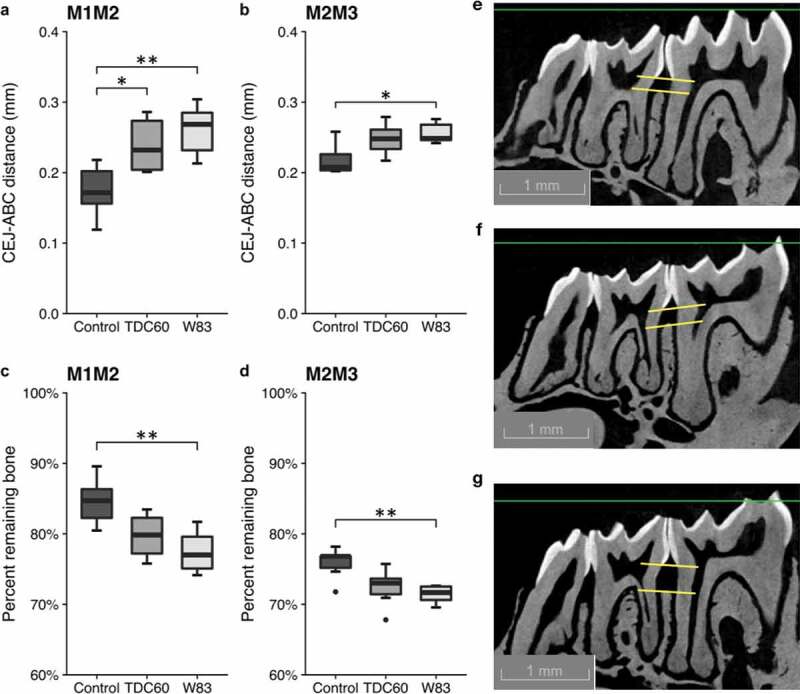
Alveolar bone loss in mice. Measurements of bone levels were made by comparing the distance from the cementoenamel junction (CEJ) to the alveolar bone crest (ABC) (**a, b**) and the percentage of remaining bone (**c, d**) at two interdental sites: between the first and second mandibular molars (M1M2) and between the second and the third mandibular molars (M2M3). Root lengths (from the CEJ to the root apex) were also measured to assess the percentage of remaining alveolar bone: Percent remaining bone (%) = ([root length – CEJ-ABC]/root length) x 100. Representative images of micro-CT of control (**e**), TDC60-treated (**f**) and W83-treated (**g**) mice are presented. Control and *P. gingivalis* infected mice were 16 weeks old (*n* = 6 mice/group). Dunn’s Kruskal–Wallis multiple comparisons, statistically significant at: *p < 0.05; **p < 0.01.

The micro-CT analysis and comparison between controls and infected mice showed that W83-treated mice had a significant alveolar bone loss (Figure 2). This was illustrated by the increase of the distance from the cementoenamel junction to the alveolar bone crest (CEJ-ABC) in both M1M2 (Figure 2a), and M2M3 interproximal sites (Figure 2b). A significant decrease of the remaining bone support was observed when the CEJ-ABC distance was related to the root length in both M1M2 (Figure 2c), and M2M3 interproximal sites (Figure 2d). While the TDC60-treated mice exhibited a trend for alveolar bone loss, only one site reached the significance threshold. The bone loss induced by *P. gingivalis* TDC60 was lower than that induced by W83 in all measurements, although no difference was found between the two groups of infected mice.

**Figure 3.**
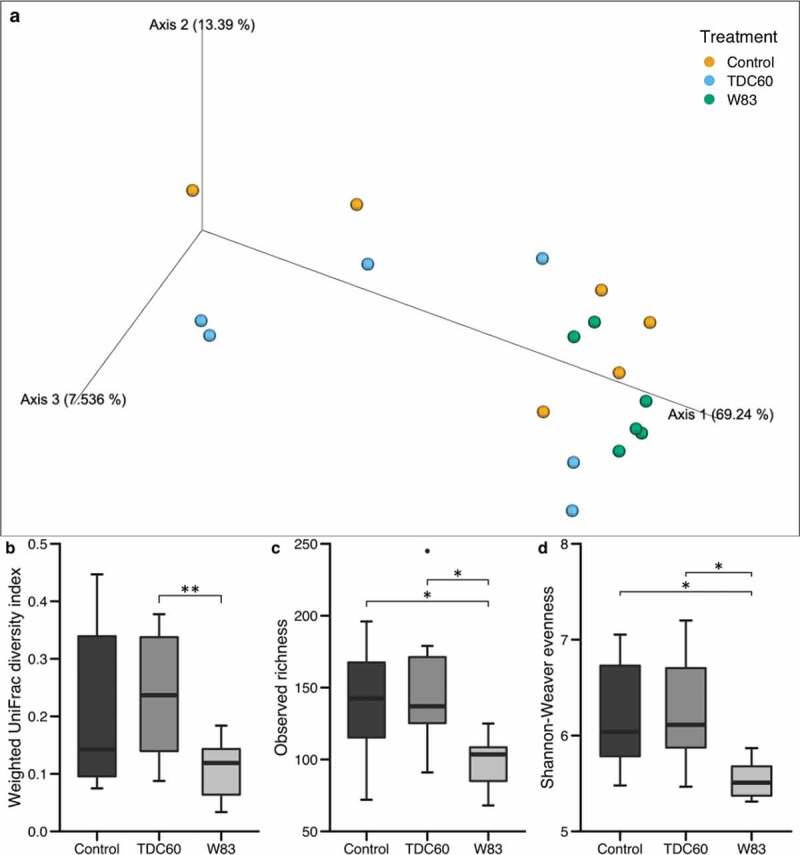
*Alpha* and *beta* diversity of the oral microbiota of mice. Bioinformatics processing of the microbiota samples from the oral cavity of control and *P. gingivalis* infected mice (*n* = 6 mice/group, 16 weeks old) allowed for Principal Coordinate Analysis (PCoA). The PCoA calculated with weighted UniFrac metric revealed clustering of W83-treated mice (**a**). Analysis of distances between samples showed significant differences between W83- and TDC60-treated mice, with both PERMANOVA and ANOSIM tests (**p < 0.01, **b**). *Alpha* diversity analysis showed significantly lower richness (S_obs_, **c**) and lower evenness (Shannon-Weaver, **d**) in W83-treated mice; Dunn’s Kruskal–Wallis multiple comparisons, statistically significant at: *p < 0.05.

During the *beta* diversity analysis of oral microbiota, the Principal Component Analysis (PCoA) revealed an apparent homogeneity in the samples from W83-treated mice (Figure 3a). Moreover, they appeared to be clustered together, on the side-lines of the two other groups, which were more distributed in the 3D exploration. The PERMANOVA and ANOSIM tests, performed on weighted UniFrac distances plotted in PCoA, showed significant differences between the samples from W83- and TDC60-treated mice (Figure 3b). Moreover, the *alpha* diversity metrics confirmed that W83-treated mice had a distinct oral microbiota, with significantly lower richness and evenness than both controls and TDC60-treated mice (Figure 3c, d).

**Figure 4.**
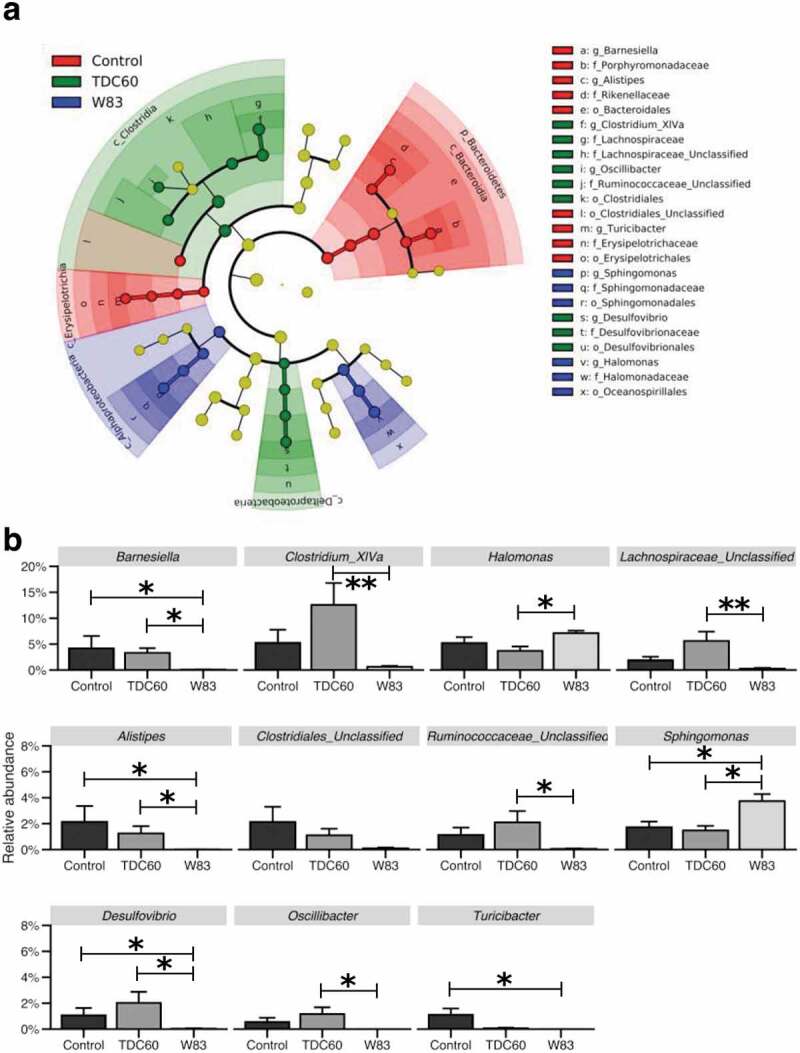
Analysis of taxa relative abundances in the oral cavity according to the treatment of mice. The linear discriminant analysis with effect size (LEfSe) cladogram identified taxa that were differentially abundant across the groups (*n* = 6 mice/group; *p* < 0.05) (**a**). Taxa at the genus-level were then filtered (excluding those with a mean relative abundance < 1% in all groups), and plotted when the Kruskal–Wallis test returned significant result (*p* < 0.05) (**b**). Dunn’s Kruskal–Wallis multiple comparisons, statistically significant at: *p < 0.05; **p < 0.01.

The taxonomic analysis of the oral microbiota showed differentially abundant taxa when assessed with LEfSe algorithm (Figure 4a). In high taxonomic levels, the samples from controls were found to be enriched in *Bacteroidetes* and *Erysipelotrichia*, while samples from TDC60-treated mice had increased abundance of *Clostridia* and *Deltaproteobacteria*. Samples from W83-treated mice had increased abundance of *Alphaproteobacteria* and *Oceanospirillales*. However, the lineages in the cladogram indicated that these variations were related with significant differences in taxa at the genus-level. These taxa were further filtered and assessed by Kruskal–Wallis and Dunn’s multiple comparisons tests (Figure 4b). As in the diversity analyses, significant variations were found between samples from W83-treated mice and the two other groups. Specifically, *Alistipes, Barnesiella, Clostridium*_XIVa, *Desulfovibrio, Oscillibacter, Turicibacter, Lachnospiraceae*_Unclassified and *Ruminococcaceae*_Unclassified were found significantly decreased in W83-treated mice; several of them were almost undetected. On the contrary, *Halomonas* and *Sphingomonas* were found significantly increased compared to controls and TDC60 groups.

## Discussion

Despite the fact that some authors inoculated mice with other species from the Socransky’s red complex [18,19], most of the studies with the periodontitis mouse model used *P. gingivalis*, which is reported as a keystone pathogen in periodontitis [20]. *P. gingivalis* TDC60 is reported to have a higher pathogenicity than *P. gingivalis* W83 [6]. However, to our knowledge, the impact of oral gavage using TDC60 has not yet been investigated in a mouse periodontitis model. Unexpectedly, despite colonization of the oral cavity of mice by both strains, TDC60 exhibited a lower ability in causing alveolar bone loss, while the oral administration of W83 initiated periodontitis in both molar interproximal sites in our experiments.

Regarding the major virulence factors of the *P. gingivalis*, they are mostly cell surface proteins such as gingipains (RgpA, RgpB and Kgp), haemagglutinins, major and minor fimbriae (FimA and Mfa1), capsule (K-antigen) and the immunodominant surface antigens RagAB (Table 1) [21]. The cysteine peptidase gingipains account for 85% of the extracellular proteolytic activity of the pathogen [22]. By degrading a variety of host proteins and taking part in the dysregulation of the host’s immune function, they are critical for bacterial colonization and the establishment of the disease [20,23]. According to a large *in silico* work, gingipains of W83 and TDC60 are similar for catalytic and C-terminal haemagglutinin domains [24]. The fimbriae are involved in both bacterial adhesion and immune subversion [25]. Moreover, the different genotypes imply various protein structures that demonstrated differential antigenicity [26]. Hence, the genomic variations of *P. gingivalis* fimbriae are reported to be related to periodontitis initiation and progression. The major fimbriae (FimA), and the fimbrial accessory proteins (FimCDE), are different between TDC60 and W83 (Table 1). Interestingly, *P. gingivalis* W83 is poorly fimbriated which could be disadvantageous; however, Baker et al. showed that a mutant *P. gingivalis* W50, deficient for the fimbrial protein (strain DPG3), could also induce bone loss [4]. In human and animal studies, type II (TDC60-like) and type IV *fimA* (W83-like) are more commonly found in periodontitis patients, and are more cytotoxic and invasive than strains with other types of fimbriae [27,28]. It is worth noting that type II *fimA* genotype is frequently reported to be more prevalent than type IV *fimA* in periodontitis-affected sites, while the latter was the most commonly found genotype among gingivitis patients [28–30]; yet, another study found that the type II was the most widely distributed genotype, among both healthy and periodontitis sites [31] suggesting that other virulence factors could be implicated and should be elucidated. *In vitro* experiments showed that the minor fimbriae (Mfa1) is implicated in coaggregation between *P. gingivalis* ATCC 33277 and *Streptococcus gordonii* [32]. Due to an insertion element, W83 is disrupted for the *mfaI* gene, while TDC60 produces Mfa1 type I [24]. This could be an advantage for colonizing the oral cavity of mice, as its indigenous microbiome is frequently dominated by *Streptococcus* [33,34]. RagA and RagB are outer membrane receptor linked to transporter TonB, and an associated lipoprotein, respectively. Serum analyses of periodontal patients revealed their immunogenicity, and RagB appeared to be an immunodominant surface antigen [35]. Four distinct sequence types (*rag-1* to *rag-4*) have been described and linked to the virulence of the bacterium. Most of the *P. gingivalis rag-1* had higher scores for virulence *in vivo* than *rag-4*, and the *rag-1* locus was associated with deep pockets in periodontitis patients [36–38]. Thus, *rag-1* could be beneficial for W83 [24]. However, studies about the virulence of the different *rag* alleles were performed prior to the isolation of TDC60, which has therefore not yet been tested. Finally, most of the *P. gingivalis* strains are encapsulated with one of the six serotypes of the capsular polysaccharide antigens (K-antigens, K1 to K6), while some strains are not encapsulated at all [39,40]. The presence of the capsule and the differences in its chemical composition imply variations in the bacterial persistence, the host’s immune responses, the adhesion and coaggregation capacities [40]. Encapsulated *P. gingivalis* strains, such as W83 (K1 [41]), have demonstrated higher virulence than non-encapsulated strains, such as ATCC 33277 (K^–^) [39,40]. However, most of the *P. gingivalis* genomes contain a similar number of capsule-related genes, irrespective of the actual presence of a capsule, indicating that these genes may be subjected to gene expression controls [5]. In summary, while Rag outer membrane proteins and capsule have not yet been characterized in TDC60, virulence mediated by FimA and Mfa1 should be in favor of TDC60. However, the impact of these proteins on the virulence is discussed in the literature and our results showed greater alveolar bone loss with W83 strain than with the TDC60 strain.

Regarding dysbiosis, a previous study showed that *P. gingivalis* triggers changes in the composition of the oral microbiota [9]. Using aerobic/anaerobic cultures, the authors have identified alterations in six different genera *spp*. Our results suggest that these alterations could be a part of a major bacterial shift, detectable with high-throughput sequencing methods. This shift, which could be linked to a dysbiosis, appeared to be strain-dependent in our diversity analyses. Indeed, only W83-treated mice exhibited a poorly diversified oral microbiota, which tended to be clustered away from the two other groups. It is noteworthy that the samples were collected 1 month after the last oral inoculation; therefore, we cannot conclude about the ability of *P. gingivalis* TDC60 to alter the microbiota during the experiments. However, the strain W83 appeared to be able to stably disrupt the host-microbiota balance in a persistent way, and it was associated with a greater alveolar bone loss.

In an interesting way, the oral microbiota of W83-treated mice exhibited very low levels of several unclassified sequences in the *Lachnospiraceae* and *Ruminococcaceae* families, both belonging to the *Clostridiales* order. Noteworthy, samples from TDC60-treated mice showed increased, but not significant, proportions of these taxa. Although they may be less numerous than previously thought, these not-yet-cultured bacteria still represent a substantial part of the normal gut microbiota in mice, as well as *Clostridium* cluster XIVa, *Barnesiella, Alistipes, Turicibacter* and *Oscillibacter* that were also depleted in W83-treated mice [42,43]. A recent study pointed out the protective role of the *Clostridia* class, that is outcompeted by *Desulfovibrio* in an experimental knocked-out mouse model of metabolic disease [44]. However, these taxa were significantly decreased in W83-treated mice. In these mice, the taxa with significantly higher proportions were *Halomonas* and *Sphingomonas*, two genera which are likely to be associated with pathological conditions. *Halomonas* was detected in lung tissue from mice with lung fibrosis [45], while *Sphingomonas spp*. are known to carry ligands for NKT cells, an important member of the innate immune defence involved in the autoimmunity process [46].

In conclusion, this study showed strain-dependent alveolar bone loss associated to a major alteration of the oral microbiota, in a mouse periodontitis model by oral gavage with *P. gingivalis*. Although described as highly pathogenic, *P. gingivalis* TDC60 had weaker consequences than W83. This could be due to their abilities to dysregulate the host’s immune responses. This study underlines the need for further clarifications about the various virulence and pathogenicity among *P. gingivalis* strains. Moreover, the lasting nature of the host-microbiota imbalance that we observed should encourage us to i) look for the occurrence of long-term deleterious inflammatory effects in various body sites of the experimental animals (gut microbiota, brain, joints, cardiovascular system, etc.), and ii) identify more precisely the *P. gingivalis* strains emerging in severe periodontitis in humans.
